# Extent of prostate cancer cases not registered in The National Cancer Register of Sweden and consequences for estimates of prostate cancer incidence and mortality

**DOI:** 10.2340/ao.v65.45596

**Published:** 2026-04-17

**Authors:** Eugenio Ventimiglia, Hans Garmo, Rolf Gedeborg, Mats Ahlberg, Armando Galdieri, Andri Wilberg Orrason, Lars Holmberg, Pär Stattin, David Robinson

**Affiliations:** a Department of Surgical Sciences, Uppsala University, Uppsala, Sweden; b Regional Cancer Center Midsweden, Uppsala University Hospital, Uppsala, Sweden; c Department of Urology, Ryhov Hospital, Jönköping, Sweden

**Keywords:** Prostate cancer, registries, incidence, mortality, PSA, ADT

## Introduction

Completeness of a cancer register reflects the proportion of incident cases captured in the population. Incomplete reporting may bias estimates of incidence, survival, and international comparisons [1]. Reporting to the Swedish National Cancer Register (NCR) is mandatory by law, yet prostate cancer (PCa) presents particular challenges. Median age at diagnosis is high and frail elderly men are often diagnosed clinically without prostate biopsy, the diagnostic gold standard [2]. In addition, prostate cancer may be listed as cause of death even when death is multifactorial due to sticky diagnosis bias [3, 4].

Very high prostate-specific antigen (PSA) levels and use of androgen deprivation therapy (ADT) are strong indicators of PCa and may identify cases not recorded in cancer registries. We aimed to quantify and characterize men with probable high-risk PCa not registered in the NCR and assess the impact on estimates of incidence and mortality.

## Patients/material and methods

We conducted a nationwide population-based cohort study using Prostate Cancer data Base Sweden (PCBaSe) Xtend, which links several mandatory national healthcare registers through the unique Swedish personal identity number. The database integrates the National Prostate Cancer Register, the NCR, the National Patient Register, the Prescribed Drug Register and the Cause of Death Register together with regional laboratory information systems containing PSA measurements and pathology data, and regional electronic medical records including in-hospital administered oncological drugs. The linkage structure and validity of PCBaSe have been described previously [5, 6].

The study population comprised men residing in 19 of the 21 Swedish healthcare regions (approximately 95% of the male population) where complete laboratory and treatment data were available during 2015–2021. Two cohorts were defined. The first consisted of men with incident prostate cancer recorded in the NCR. The second consisted of men without a prostate cancer registration but with strong clinical evidence of disease based on either treatment with ADT or markedly elevated prostate-specific antigen (PSA > 50 ng/mL).

To reduce false positive classification, PSA elevations likely attributable to infection were excluded using predefined criteria based on antibiotic treatment patterns and rapid PSA decline. In addition, men with a recorded negative prostate biopsy were not considered cases (see Supplementary material for details).

Date of diagnosis was defined at first dispensed ADT prescription or 180 after initial PSA test > 50 ng/ml if no ADT prescriptions were observed in this period. ADT exposure included gonadotropin-releasing hormone agonists or antagonists, anti-androgens, and androgen receptor pathway inhibitors.

Information on comorbidity was obtained from the National Patient Register using a 10-year look-back period and from dispensed medications during the preceding year. These data were combined to calculate health-adjusted life expectancy using previously validated Swedish models [7, 8]. Vital status and cause of death were obtained from the Cause of Death Register.

Participants were followed from date of diagnosis until death or 31 December 2021. For men registered in the NCR, cumulative incidence of prostate cancer death and death from other causes was estimated treating competing events accordingly. For men not registered, subsequent registration in the Cancer Register, prostate cancer death, and death from other causes were treated as competing events. A man who was not registered in the NCR at diagnosis, but was later registered in NCR, contributed to both cohorts.

We then stratified the cohort of men with Pca in NCR according to T stage, M stage, and life expectancy, in order to identify men in NCR with similar survival as the men not in NCR. Based on the 8-year cumulative incidence of events for men not in NCR, we estimated cutoffs for life expectancy for men in NCR with metastatic disease and locally advanced disease (T stage T3, T4) in order to assess their 8-year mean survival. Finally, we evaluated the impact of non-registered cases on population-level incidence and mortality by recalculating absolute and relative risk estimates after inclusion of these cases.

## Results

Between 2015 and 2021, 69,422 men were registered with PCa in the NCR and 3,143 men fulfilled criteria for probable PCa but were not registered.

Including unregistered men increased the average annual number of PCa cases from 9,920 to 10,370 (+4.3%), a stable increase over time (~5% annually, Table 1).

**Table 1:** Annual number of men with prostate cancer identified by elevated PSA values or use of androgen deprivation therapy (ADT) and number of men registered in The National Cancer Register of Sweden with a prostate cancer diagnosis.

Year of diagnosis	Prostate cancer cases not registered in The National Cancer Register of Sweden	Prostate cancer cases in The National Cancer Register of Sweden	% Non-registered prostate cancer cases
2015	469	10,063	4.5
2016	507	10,183	4.7
2017	429	9,957	4.1
2018	429	10,512	3.9
2019	419	10,686	3.8
2020	436	8,761	4.7
2021	454	9,260	4.7

Men with Pca not registered in NCR were older, median age of 87 years (inter quartile range, [IQR]: 81–91) compared with 70 years (IQR: 64–76) among those registered in NCR. Their median health adjusted life expectancy was 4 years (IQR: 3–6), compared with 15 years (IQR: 10–19) among men registered in NCR (Supplementary Table 1).

**Figure 1:**
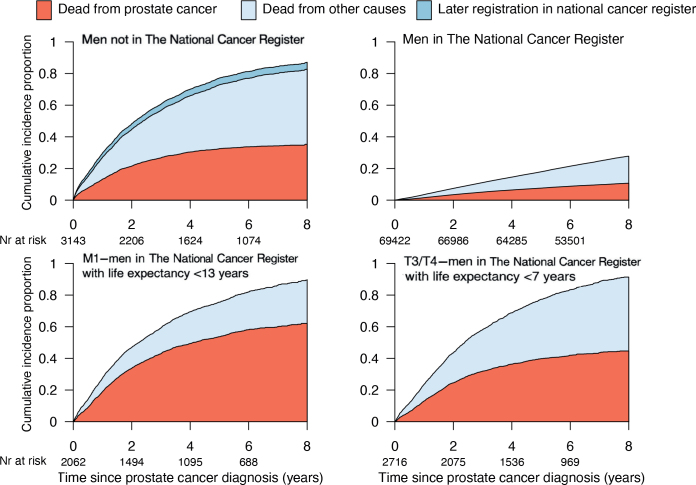
Cumulative incidence of death from prostate cancer and other causes for men registered or not registered in The National Cancer Register.

Eight years after diagnosis, men not in NCR had higher cumulative incidence of Pca death (35% vs. 11%) and death from other causes (48% vs. 17%), compared to men in NCR (Figure 1). Since staging information was not available for men not in the NCR, we tried to find a group of men in NCR who had similar survival as men with Pca not in NCR, based on tumor stage and life expectancy. Men in NCR with metastases and < 13 years of health-adjusted life expectancy and men with stage T3-4 and < 7 years of health-adjusted life expectancy had largely similar 8-year survival as men not in NCR (Figure 1).

The absolute 5-year Pca mortality risk increased with 1.2 percentage points when men not registered in the NCR were included in the estimation, corresponding to a 15% increase. The overall survival increased with 2.4 percentage points, corresponding to a 13% increase (Supplementary Figure 1).

## Discussion and conclusion

Despite legally mandated reporting, approximately 4–5% of prostate cancers were not captured by the Swedish NCR. These missing cases represent a clinically distinct population: very elderly men with limited life expectancy and advanced disease who were rarely biopsied. Their diagnoses appear to have been made clinically and therefore not always reported from pathology departments, which are central sources for cancer registration in Sweden.

The impact on incidence was modest but consistent, whereas mortality estimates were slightly more affected because the unregistered population had substantially worse survival. This may influence interpretation of registry-based prostate cancer mortality and international comparisons, but in general its consequences are limited [1, 9–14].

Comparable Nordic registries achieve higher completeness partly through inclusion of death certificate cases and hospital discharge diagnoses [15, 16]. Our findings suggest such approaches would identify a substantial proportion of missing cases. Strengths include nationwide linkage of laboratory, prescription, and registry data enabling identification of probable prostate cancer outside traditional reporting pathways thanks to very elevated PSA values and use of ADT.

## Conclusion

Approximately 4–5% of prostate cancers in Sweden were not registered in the NCR. These patients were substantially older, had shorter life expectancy, and higher mortality. Inclusion of these cases increased estimated prostate cancer mortality by ~15% but had limited effect on incidence. Incomplete capture mainly reflects clinically diagnosed advanced cancer in frail elderly men and should be considered when interpreting registry-based comparisons and in the research setting.

## Supplementary Material



## Data Availability

Data used in this study were extracted from the Prostate Cancer data Base Sweden (PCBase), which is based on the National Prostate Cancer Register (NPCR) of Sweden and linkage to several national health-data registers. The data cannot be shared publicly because the individual-level data contain potentially identifying and sensitive patient information and cannot be published due to legislation and ethical approval (https://etikprovningsmyndigheten.se). Use of the data from national health-data registers is further restricted by the Swedish Board of Health and Welfare (https://www.socialstyrelsen.se/en/) and Statistics Sweden (https://www.scb.se/en/) which are Government Agencies providing access to the linked healthcare registers. The data will be shared on reasonable request in an application made to any of the steering groups of NPCR and PCBase (contact npcr@npcr.se). To request data or analytic code from this study, contact the corresponding author. For detailed information, please see www.npcr.se/in-english, where registration forms, manuals, and annual reports from NPCR are available alongside a full list of publications from PCBase.
