# Supplementary material for: Extent of prostate cancer cases not registered in The National Cancer Register of Sweden and consequences for estimates of prostate cancer incidence and mortality
Source: Acta Oncol. 2026 Apr 17;65:45596. doi: 10.2340/ao.v65.45596 (PMC13097090; doi:10.2340/ao.v65.45596)
Supplement: Supplementary file 1 [file AO-65-45596-s1.pdf]

Supplementary material has been published as submitted. It has not been copyedited, or typeset by Acta Oncologica

Supplementary material

Definition of PCa diagnosis based on filled prescriptions of androgen deprivation therapy (ADT)

We retrieved filled prescriptions of ADT in The National Prescribed Drug Register (7) and from regional electronic patient record systems. Specifically, for each recorded PSA value in men above age 30 without a PCa diagnosis in NCR, we extracted all filled prescriptions for:

a) Gonadotropin-releasing hormone antagonist (GnRH, ATC codes L02AE01, L02AE02, L02AE03, L02AE04, L02AE05)

b) Degarelix (ATC code L02BX02)

c) First generation antiandrogens (ATC codes L02BB01, L02BB02, L02BB03)

d) Androgen receptor pathway inhibitors (ARPIs, ATC codes L02BX03, L02BB04-6)

If any filled prescriptions of these medications were found during the 180 days following the PSA test, the date of Pca diagnosis was set to the earliest date of filling of this prescription.

Definition of prostate cancer based on elevated PSA values

We identified all PSA measurements above 50 ng/mL and searched for the following events up 180 days after date of the elevated PSA:

a) PCa diagnosis in the NCR

b) Filled prescriptions for ADT

c) A negative prostate biopsy

d) Indications of a likely urinary tract infection:

   i. Treatment with ciprofloxacin (ATC-code J01MA02) within 28 days and PSA-half time ≤90 days, or
   ii. Treatment with ciprofloxacin within 14 days and no further PSAs taken.

If none of these events were identified, then the date of Pca diagnosis was set to 180 days after the date of the PSA value.

Prostate biopsy

In men not registered in NCR but identified as having Pca we confirmed if they had undergone prostate biopsies in the 180-day periods preceding and following the diagnosis date, by using linkage to pathology laboratory information systems available in PCBase Xtend.

Supplementary Figure 1: Risk of death from prostate cancer and any cause for men registered or not registered in The National Cancer Register

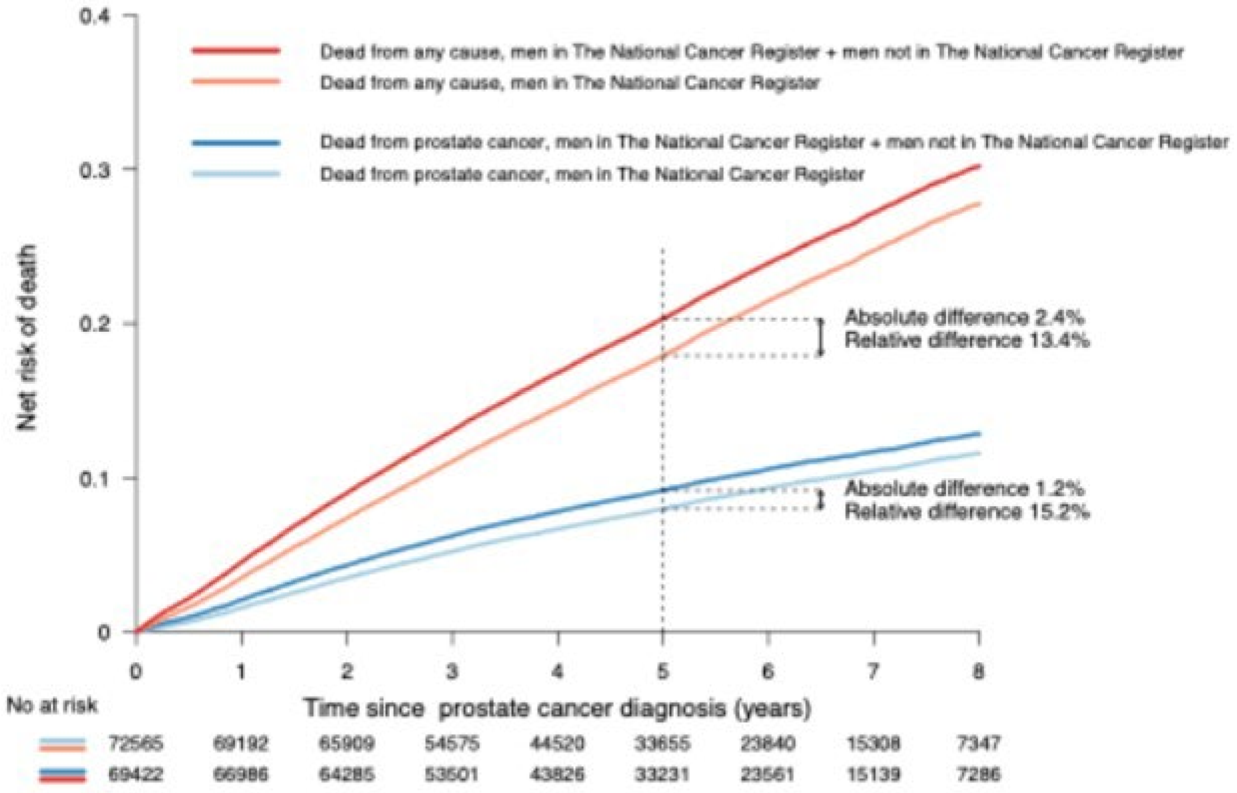

Supplementary Table 1: Characteristics of men registered or not registered in the National Cancer Register of Sweden. *M1 and ICD code C61 for prostate cancer (Pca) at the last visit prior to diagnosis.

	Prostate cancer cases not registered in The National Cancer Register identified by elevated PSA values (n=1214)		Prostate cancer cases not registered in The National Cancer Register identified by use of androgen deprivation therapy (n=1929)		Prostate cancer cases in The National Cancer Register (n=69 422)	
**Age, years**						
**Median (Q_1-Q_3)**	86.5	(80.8–90.6)	84.7	(77.8–89)	70.0	(64.0–76.0)
**n (%)**						
≤80	272	(22)	567	(29)	60 852	(88)
81-85	225	(19)	387	(20)	5575	(8)
86-90	361	(30)	529	(27)	2413	(3)
91+	356	(29)	378	(20)	580	(1)
**Life expectancy, years**						
**Median (Q_1-Q_3)**	4.1	(2.8–6.1)	5.2	(3.3–8.0)	14.8	(10.5–19.4)
**n (%)**						
≤3	353	(29)	399	(21)	943	(1)
3.01-4.5	325	(27)	355	(18)	1818	(3)
4.51-7	296	(24)	514	(27)	4663	(7)
7.01+	240	(20)	593	(31)	61 996	(89)
**Record of M1 and Pca in Patient Register*, n (%)**						
M1 and Pca	106	(9)	206	(11)	551	(1)
M1 not Pca	15	(1)	52	(3)	362	(1)
Not M1 but Pca	280	(23)	628	(33)	1000	(1)
Not M1 not Pca	671	(55)	875	(45)	46 596	(67)
No record	142	(12)	168	(9)	20 913	(30)
**Charlson Comorbidity Index, n (%)**						
0	991	(82)	1616	(84)	62 937	(91)
1	69	(6)	84	(4)	2392	(3)
2	49	(4)	85	(4)	2282	(3)
3	42	(3)	45	(2)	854	(1)
4+	63	(5)	99	(5)	957	(1)
**T stage, n (%)**						
T1					33 164	(48)
T2					21 043	(30)
T3					8849	(13)
T4					1661	(2)
Tx					3177	(5)
**N stage, n (%)**						
N0					14 913	(21)
N1					1422	(2)
Nx					53 087	(76)

**M stage n (%)**	
M0	64610 (93)
M1	1691 (2)
Mx	3121 (4)

***M1 as for ICD10 code C77-80. ICD10 code C61 for prostate cancer (Pca) at the last visit prior to diagnosis.**
